# Ethnomedicinal Practices of the Fabaceae Family in Tanzania: A Systematic Review

**DOI:** 10.1155/tswj/6918847

**Published:** 2026-03-20

**Authors:** David Sylvester Kacholi

**Affiliations:** ^1^ Department of Biological Sciences, Dar es Salaam University College of Education (DUCE), Dar es Salaam, Tanzania

**Keywords:** ethnobotany, ethnomedicine, Fabaceae, herbal remedies, natural compounds, pharmacological activities, traditional knowledge

## Abstract

This study is aimed at reviewing the ethnomedicinal, phytochemical and pharmacological properties of Fabaceae species used as sources of traditional medications in Tanzania. Using the Systematic Reviews and Meta‐Analyses (PRISMA) methodology, a comprehensive review was conducted on Fabaceae species used by Tanzanians through electronic databases such as Web of Science, Scopus, Google Scholar, African Journals Online, PubMed and Wiley Online Library. After systematic screening and eligibility assessment, 51 studies highlighted diverse traditional healing practices across the country′s regions. The botanical names were verified through the Plants of the World Online database. The study revealed that 97 Fabaceae species traditionally treat human ailments in Tanzania. The genera with the highest number of species with medicinal uses are *Vachellia*, followed by *Albizia* and *Senegalia*. The species with the highest usage records are *Abrus precatorius*, *Cassia abbreviata*, *Tamarindus indica*, *Alantsilodendron pilosum* and *Erythrina abyssinica*. Trees (46%), herbs (30%) and shrubs (24%) are the primary sources of traditional medicines, whereas roots (63%), leaves (41%) and stem bark (28%) are the most widely used plant parts. The majority of the species are characterized by flavonoids (62.5%), followed by phenolic compounds (38.9%), terpenoids (33.3%) and tannins (33.3%). Though many Fabaceae species have been evaluated for their phytochemical and pharmacological properties, validating their toxicological properties is paramount.

## 1. Introduction

The Fabaceae family, commonly referred to as the legume, bean, or pea family, is a captivating subject of study, primarily due to its significant ethnopharmacological value. It is recognised as the second‐largest plant family in terms of species richness among flowering plants [1]. Fabaceae exhibits a wide geographical distribution across various ecological habitats, following the Poaceae family. This family is essential for maintaining ecological balance, supporting agricultural practices, and sustaining the livelihoods of numerous communities [2]. In Tanzania, medicinal plants (MPs) from the Fabaceae family play a vital role due to their nitrogen‐fixing capabilities, which enhance soil fertility and provide food sources, timber, and medicinal benefits. The country′s unique climatic and geographical conditions nurture a variety of leguminous plants, creating distinct ecological niches that support diverse species [3]. Recent morphological and molecular studies have further confirmed that this family is monophyletic. Comprising trees, shrubs, herbs, climbers and aquatic species, its diversity contributes to its remarkable nature [2, 4].

The Fabaceae family includes many species of significant traditional and economic importance worldwide. These plants serve as sources of traditional medicines, timber, food, fodder, ornamental plants, dyes, fibres, fuels, gums and insecticides [5]. Research reviews from various countries have highlighted the crucial role that Fabaceae species play in providing ecosystem services and resources vital for human well‐being and survival [5, 6]. Numerous members of this family have been the focus of extensive studies regarding their bioactive chemical constituents, including phenolic acids, flavonoids, lectins, saponins, alkaloids and carotenoids. Pharmacological investigations have shown that certain species display potent anticancer, antioxidant, antimicrobial, anti‐inflammatory, analgesic, antiulcer, antidiabetic, antirheumatic, cytotoxic and antiparasitic properties [7, 8]. Consequently, comprehensive phytochemical and pharmacological evaluations of these valuable Fabaceae species may pave the way for the discovery and development of novel pharmaceutical products, functional food ingredients, and cosmetic applications [5]. Despite the identification of various secondary metabolites within the Fabaceae family, it has received relatively little attention in ethnopharmacological research.

This study was conducted to systematically investigate and document the ethnomedicinal knowledge associated with Fabaceae species in Tanzania. The synthesis reveals gaps in understanding the therapeutic potential of these legumes and highlights important areas within ethnopharmacological research that require further exploration. The implications of this research are profound, as it may uncover new medicinal applications and facilitate the development of innovative pharmaceutical products, thus advancing the field and enhancing human health outcomes. This emphasises the study′s critical significance and its contributions to the discipline of ethnopharmacology.

## 2. Methodology

### 2.1. Literature Search Strategy

**Figure 1:**
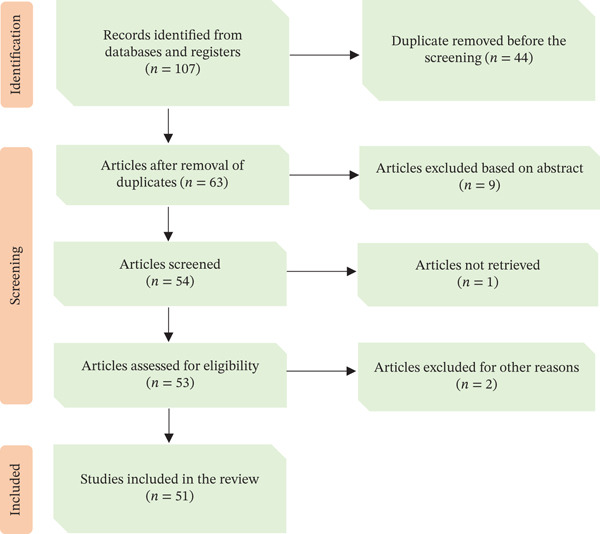
PRISMA flow diagram demonstrating the identification and screening of the records used in the review process.

A comprehensive literature search on MPs of the Fabaceae family used as TMs in Tanzania was conducted between August 2023 and October 2024. Data were obtained from online databases, including Google Scholar, Web of Science, BioMed Central, PubMed, SpringerLink, Science Direct, Scielo, Scopus, African Journals Online (AJOL) and JSTOR. Books, book chapters, scientific reports, theses, and dissertations were also retrieved from the University of Dar es Salaam (UDSM) libraries. Terms and keywords such as Tanzania, MPs, traditional medicine, ethnomedicine, ethnobotany, indigenous, ethnopharmacology, phytomedicine, medicinal Fabaceae and medicinal Leguminosae were used to identify relevant articles, as shown in the PRISMA flowchart (Figure 1). This rigorous and systematic approach ensures the reliability and validity of the findings, instilling confidence in the research process and its outcomes while providing a solid foundation for future studies.

### 2.2. Inclusion Criteria

The review included both published and unpublished ethnobotanical studies reporting on MPs in Tanzania. Only original research articles written in English were considered. From each study, information was extracted on the scientific names of the MPs, their growth forms, the plant parts used, and ailments treated. Botanical nomenclature was verified, and missing details such as growth habits and life forms were supplemented using the Plant of the World Online database (https://powo.science.kew.org/).

### 2.3. Exclusion Criteria

Review studies and those lacking essential ethnobotanical information, such as scientific names, plant parts used, routes of administration or study locations were excluded from the analysis. Likewise, research focusing solely on livestock, publications with restricted access, abstracts without full texts, non–open‐access sources, studies conducted outside Tanzania and articles not published in English were excluded.

### 2.4. Data Analysis

Descriptive statistical methods were employed to analyse and summarise the obtained data. The results are presented as percentages and frequencies and are therefore depicted in tables, bar charts and pie charts.

## 3. Results and Discussion

### 3.1. MP Diversity

This review Ps from the Fabaceae family that are habitually used for managing and treating various human ailments in Tanzania (see Table 1). Among these, 86.5% (84 species) are native, whereas 13.5% (12 species) are exotic, found in the wild or cultivated ecosystems for food, forage, and ornamental purposes. A similar ethnobotanical investigation conducted in Zimbabwe revealed that 101 species from the Fabaceae family are utilised for medicinal purposes [1]. Likewise, research by Van Wyk et al. [2] discovered that 19.3% (338 species) of the 1748 Fabaceae members serve as herbal remedies in Southern Africa. Comparable results have been reported among Thailand′s marginalised Karen ethnic group, where 37.9% (261) of the 688 Fabaceae species are employed for medicinal uses [3]. The importance of the family in traditional pharmacopoeias worldwide may be attributed to the widespread distribution of its members, which thrive in diverse habitats and climates, ensuring their availability throughout all seasons [4, 5].

**Table 1:** List of medicinal Fabaceae species of Tanzania.

Scientific name	Habit	Source	Part used	Medicinal uses	Literature records	References
*Abrus precatorius* L.	H	W	R. L, Sb, Fr	Diarrhoea, eye inflammation, women′s infertility, aphrodisiac, wounds, sores, cough, malaria, asthma, urinary transmitted infection, erectile dysfunction, gonorrhoea, snakebite, epilepsy and oral candidiasis	19	[9, 10]
*Afzelia quanzensis* Welw.	T	W, C	R	Malaria and snakebite	3	[11, 12]
*Aganope stuhlmannii* (Taub.) Adema	T	W	R	Stomach ache and sickle cell	1	[11, 13]
*Alantsilodendron pilosum* Villiers ^∗^	T	W	R, L, Sb	Asthma, wounds, boils, abscesses, malaria, infertility, erectile dysfunction, cough, snakebite, and venereal diseases	11	[8, 10, 11, 14–18]
*Albizia anthelmintica* (A. Rich.) Brongn.	S	W	R, L	Malaria, infertility and asthma	7	[8, 10–12, 16, 19, 20]
*Albizia gummifera* (J.F. Gmel.) http://C.A.Sm.	T	W	Sb	Malaria and stomach ache	3	[11, 21, 22]
*Albizia harveyi* E. Fourn.	S	W	L, R	Abscesses, wounds, infertility, dental problems, libido, scabies and skin diseases	4	[8, 11, 14, 23]
*Albizia lebbeck* (L.) Benth ^∗^	T	W	L, Fr	Malaria, stomach ache and worms	2	[11]
*Albizia petersiana* (Bolle) Oliv.	T	W	R, Sb	Skin diseases	1	[9]
*Albizia schimperiana* Oliv.	T	W	R	Hypertension and postpartum stomach pains	1	[24]
*Albizia verrucosa* Capuron	T	W	R	Stomach ache and worms	1	[11]
*Albizia versicolor* Welw. ex Oilv.	T	W	R, Sb	Skin diseases and Boils	1	[9]
*Alysicarpus ovalifolius* (Schumach.) J. Léonard	H	W	L	Fractured borne	1	[11]
*Arachis hypogaea* L.	H	C	Se	Dysentery, gastric ulcer and urinary tract infections	2	[13, 25]
*Bauhinia tomentosa* Vell.	H	W	R	Constipation and Stomach ache	1	[11]
*Bobgunnia madagascariensis* (Desv.) J.H. Kirkbr. and Wiersema	T	W	R	Malaria and yellow fever	1	[24]
*Brachystegia boehmii* Taub.	T	W	L	Snakebite	1	[17]
*Brachystegia spiciformis* Benth.	T	W	Sb	Snakebite	1	[17]
*Burkea africana* Hook.	T	W	Sb	Headache	1	[24]
*Cajanus cajan* (L.) Huth ^∗^	S	C	R, L, Se	Infertility, diarrhoea, nausea, swelling of legs, diabetes mellitus, and oral candidiasis	8	[9, 10, 13, 17, 26–29]
*Cassia abbreviata* Oliv.	T	W	R, L, Sb	Malaria, fever, sores, infertility, stomach ache, erectile dysfunction, gonorrhoea, syphilis, pneumonia, snakebite, respiratory bacterial infections, toothache, abdominal pains, back pains, feet pains, diabetes mellitus and skin infections	19	[14, 15, 17–19, 30–33]
*Cassia burttii* Baker f.	S	W	R	Amenorrhoea	1	[34]
*Chamaecrista absus* (L.) H.S. Irwin and Barneby	H	C	L	Asthma	2	[16, 34]
*Chamaecrista mimosoides* (L.) Greene	H	W	L, R, Sb	Asthma, mental illness, cough and tuberculosis	2	[9, 27]
*Dalbergia malangensis* E.C. Sousa ^∗^	H	W	L	Malaria	1	[35]
*Dalbergia melanoxylon* Guill. and Perr.	T	W	R, Tw	Rashes, abscesses, infertility, stomach ache and snakebite	5	[13, 14, 17, 22, 36]
*Dalbergia nitidula* Baker	S	W	L	Constipation	1	[11]
*Delonix elata* (L.) Gamble	S	W	L	Asthma and cough	1	[32]
*Dolichos kilimandscharicus* Taub.	H	C	L	Malaria, gonorrhoea and syphilis	1	[26]
*Dolichos oliveri* Schweinf.	H	W	R	Infertility	1	[17]
*Entada abyssinica* Steud. ex A. Rich.	T	W	R, L, Sb	Infertility, menorrhagia, gonorrhoea, colds, syphilis, coughs, charms, respiratory bacterial infections and diabetes mellitus	9	[8, 11, 17, 27, 29, 31, 32, 37, 38]
*Entada leptostachya* Harms	H	W	R	Tuberculosis, skin rashes and herpes	1	[27]
*Eriosema parviflorum* E. Mey	H	W	L	Malaria	1	[35]
*Eriosema psoraleoides* (Lam.) G. Don.	S	W	R, L	Malaria and Chronic diarrhoea	1	[27, 39]
*Erythrina abysinica* Lam.	T	W	F, Sb, R	Malaria, haemorrhoids, asthma, snakebite, stomach ache, skin rashes, herpes zoster, herpes simplex and tuberculosis	8	[11, 17–19, 26, 27, 32, 35, 40]
*Erythrina haerdii* Verdc.	T	W	Sb	Malaria	1	[41]
*Erythrina sacleuxii* Hua	T	W, C	Sb, R, L	Malaria	2	[30, 35]
*Erythrina schliebenii* Harms	T	W, C	Sb	Malaria	1	[35]
*Faidherbia albida* (Delile) A. Chev.	T	W, C	R	Infertility	1	[17]
*Guilandina volkensii* (Harms) G.P. Lewis	S	W, C	L	Malaria	1	[34]
*Indigofera arrecta* Hochst ex A. Rich.	H	W	Tw	Malaria and snakebite	3	[11, 35, 42]
*Indigofera drepanocarpa* Taub.	H	W	R	Malaria	1	[43]
*Indigofera lupatana* Baker f.	H	W	L	Asthma	2	[16, 22]
*Indigofera rhynchocarpa* Welw. ex Baker	H	W	R	Malaria	1	[20]
*Indigofera suffruticosa* Mill. ^∗^	S	C	R	Urinary transmitted infections	1	[44]
*Indigofera swaziensis* Bolus	S	W	R	Diarrhoea	1	[19]
*Indigofera volkensii* Taub.	H	W	L	Urinary tract infection and gonorrhoea	1	[11]
*Isoberlinia angolensis* (Welw. ex Benth.) Hoyle and Brenan	T	W	Sb	Wounds and snakebite	2	[14, 17]
*Julbernardia globiflora* (Benth.) Troupin	T	W	R	Spinal cord pain, hernia, joint pain, infertility and stomach ache	1	[11]
*Kotschya africana* Endl.	H	W	Tw	Malaria and spinal cord numbness	2	[11, 35]
*Lonchocarpus capassa* Rolfe	T	W	Wh	Snakebite	2	[22, 24]
*Macrotyloma axillare* (E. Mey) Verdc.	H	W	Tw	Malaria	1	[35]
*Millettia lasiantha* Dunn	H	W	Sb	Diarrhoea	1	[19]
*Millettia usaramensis* Taub.	T	W	R	Snakebite	1	[22]
*Mucuna pruriens* (L.) DC	H	W	R, Se	Male infertility	1	[9]
*Mundulea sericea* (Willd.) A. Chev.	T	W	R	Erectile dysfunction and stomach ache	2	[13, 15]
*Ormocarpum kirkii* S. Moore	S	W	Wh	Against evil spirits	1	[11]
*Ormocarpum trachycarpum* (Taub.) Harms	S	W	L	Snakebite	1	[17]
*Pericopsis angolensis* (Baker) Meeuwen	T	W	L, Sa	Sores, burns, gonorrhoea, syphilis, hernia and dysentery,	4	[9, 13, 14, 37]
*Philenoptera bussei* (Harms) Schrire	T	W	R, Sb	Infertility, menorrhagia, Increase volume of milk to lactating mother and allergy	4	[8, 11, 24, 45]
*Piliostigma thonningii* (Schumach.) Milne‐Redh.	T	W	R, Fr, Sb	Infertility, menorrhagia, cough, snakebite, stomach ache, libido disorder and anaemia	6	[11, 17, 32, 34, 46, 47]
*Pleurolobus salicifolius* (Poir.) H. Ohashi and K. Ohashi	H	C	Tw, L	Malaria	1	[35]
*Pterocarpus angolensis* DC.	T	W	R, Sb, Sa	Infertility, wounds, gonorrhoea, snakebite and anaemia	5	[11, 14, 17, 20, 37]
*Pseudovigna argentea* (Willd.) Verdc.	H	W	L	Vaginal condidiasis	1	[10]
*Pterocarpus tinctorius* Welw.	T	W	Sb	Haemorrhoids, diarrhoea, heartburn and dysentery	3	[13, 28, 40]
*Senegalia polyacantha* (Willd.) Seigler and Ebinger	T	W	R, Sb	Malaria	1	[17]
*Senegalia brevispica* (Harms) Siegler and Ebinger	T	W	R	Menorrhagia, snakebite and dysmenorrhoea	2	[11, 22]
*Senegalia mellifera* (Vahl) Siegler and Ebinger	T	W	R, L, Sb	Amenorrhoea, infertility and erectile dysfunction	5	[8, 15, 17, 45, 48]
*Senegalia nigrescens* (Oliv.) P.J.H. Hurter	T	W	R	Infertility	1	[17]
*Senegalia pentagona* (Schumach.) Hook. f.	T	W	R	Amenorrhoea	1	[8]
*Senegalia polyacantha* (Willd.) Seigler and Ebinger	T	W	R	Asthma, infertility and snakebite	4	[16, 20, 22, 49]
*Senegalia senegal* (L.) Britton	T	W	R	Abscesses and erectile dysfunction	2	[14, 15]
*Senna alata* (L.) Roxb. ^∗^	S	W	L	Malaria, snakebite and dysmenorrhoea	3	[11, 34, 39]
*Senna didymobotrya* (Fresen.) H.S. Irwin and Barneby	S	W	L, R	Malaria, diarrhoea, oedema and vomiting	4	[11, 30, 33, 35]
*Senna obtusifolia (L.)* H.S. Irwin and Barneby ^∗^	H	W	L	Malaria	1	[30]
*Senna occidentalis* (L.) Link ^∗^	S	W	L, R	Malaria, menorrhagia, snakebite, hernia, chronic diarrhoes and dysmenorrhoea	3	[8, 11, 26, 27]
*Senna siamea* (Lam.) H.S. Irwin and Barneby ^∗^	T	W, C	L, R	Malaria, gonorrhoea, stomach ache, diarrhoea, sexually transmitted diseases and typhoid	4	[11, 13, 37, 50]
*Senna singueana* (Delile) Lock	T	W	L, R	Malaria, erectile dysfunction, wounds, tapeworms and diabetes mellitus	5	[14, 15, 24, 31, 33]
*Sesbania microphylla* Harm.	S	W	L	Malaria	1	[26]
*Sesbania sesban* (L.)Merr.	S	W	R	Stomach ache and worms	1	[11]
*Tamarindus indica* L. ^∗^	T	C	R, L, Sb, Fr	Malaria, wounds, constipation, infertility, erectile dysfunction, diarrheal, vaginal candidiasis, foot pain and urinary tract infections	13	[11, 13–15, 17, 19, 24, 28, 31, 46, 51–53]
*Tephrosia aequilata* Baker	H	W, C	Wh	Malaria	1	[35]
*Tephrosia luzoniensis* Vogel ^∗^	H	W	L	Cough and flu	1	[11]
*Tephrosia purpurea* (L.) Pers.	H	W, C	R	Asthma	2	[16, 22]
*Tephrosia vogelii* Hook.f. ^∗^	H	W	Sb	Insecticide	1	[9]
*Vachelia tortilis* (Forssk.) Galasso and Banfi	S	W	R	Infertility	1	[20]
*Vachellia drepanolobium* (Harms ex Y. Sjöstedt) P.J.H. Hurter	S	W, C	R	Abscesses	1	[14]
*Vachellia gerrardi* (Benth.) P.J.H. Hurter	S	W	R	Diarrhoea	1	[19]
*Vachellia hockii* (De W.) Seigler and Ebinger	S	W	R, B	Abscesses and herpes zoster	1	[14, 27]
*Vachellia nilotica* (L.) P.J.H. Hurter and Mabb	T	W	R, Sb, L	Malaria, asthma, cough, blood pressure, stomach ache, libido disorder, hemodialysis, gonorrhoea, fungal diseases and hernia	6	[10, 11, 16, 20, 24, 32]
*Vachellia robusta* (Burch.) Kayl. and Boatwr.	S	W	R, Sb	Dysmenorrhoea, libido disorder, spinal cord and joint numbness	1	[8, 11]
*Vachellia seyal* (Delile) P.J.H. Hurter	T	W, C	R	Malaria	1	[11]
*Vachellia sieberiana* (DC.) Kyal. and Boatwr.	T	W	R	Malaria	1	[11]
*Vachellia zanzibarica* (S. Moore) Kyal. and Boatwr.	S	W	R, Sb	Constipation and candida infections	2	[10, 11]
*Vigna radiata* (L.) R. Wilczek	H	W	Se	Snakebite	1	[54]
*Vigna unguiculata* (L.) Walp.	H	C	R, L, Wh	Cough, chest pain, abscess, gonorrhoea, malaria, hernia and anaemia	5	[8, 9, 37, 47, 55]
*Zenkerella grotei* (Harms) J. Léonard	T	W	L	Diarrhoea	1	[19]

*Note:* The asterisk denotes exotic.

Abbreviations: AP, aerial parts; C, cultivated; Fr, fruit; H, herb; L, leaf; R, root; RB, root bark; S, shrub; Sa, sap; Sb, stem bark; Se, seed; T, tree; W, wild; Wh, whole plant.

*Vachellia* has the highest number of MPs (nine species), followed by *Albizia* (eight species), *Senegalia* and *Indigofera* (each with seven species), *Senna* (six species), *Tephrosia* (four species), and *Dalbergia* and *Erythrina* (each with three species). However, the genus with the most literature records is *Senna* (18 records), followed by *Abrus* and *Cassia* (17 records each), *Tamarindus* (13 records), *Senegalia* (11 records), *Erythrina* and *Indigofera* (10 records each), and *Vachellia* (nine records) (Table 1). The findings align with those of Arnold et al. [56], who recorded 32 species of *Vachellia* and Indigofera, 32 species of *Senna*, and 17 species of *Albizia*, all used for medicinal purposes in Southern Africa. Similarly, a study in Botswana found that *Vachellia* (six species), followed by *Indigofera* (five species) and *Albizia* (three species), are the most utilised genera in the country′s traditional medicines [57]. Furthermore, several species of Vachellia, *Senegalia, Senna, Indigofera, Erythrina,* and *Albizia* are detailed in the monograph MPs of South Africa [2] with respect to their botany, medicinal uses, preparation, dosage, active ingredients, and pharmacological effects.

The species with most records in the surveyed literature are *Abrus precatorius* L. and *Cassia abbreviata* Oliv. (19 records each), followed by *Tamarindus indica* L. (13 records), *Alantsilodendron pilosum* Villiers (11 records), *Entada abyssinica* Steud. ex A. Rich (nine records), and *Erythrina abyssinica* Lam. (eight records), *Cajanus cajan* (L.) (eight records), *Albizia anthelmintica* (A. Rich) Brongn. (seven records), *Vachellia nilotica* (L.) P.J.H. Hurter and Mabb (seven records), *Piliostigma thonningii* (Schumach.) Milne‐Redh. (six records), and Huth, *Dalbergia melanoxylon* Guill. & Perr, *Pterocarpus angolensis* DC, *Senegalia mellifera* (Vahl) Siegler and Ebinger, *Senegalia polyacantha* (Willd.) Seigler and Ebinger, and *Senna singueana* (Del.) Lock (five records each). The remaining MPs had, at most, five literature records (Table 1).

### 3.2. Growth Habit, Parts Used and Sources

**Figure 2:**
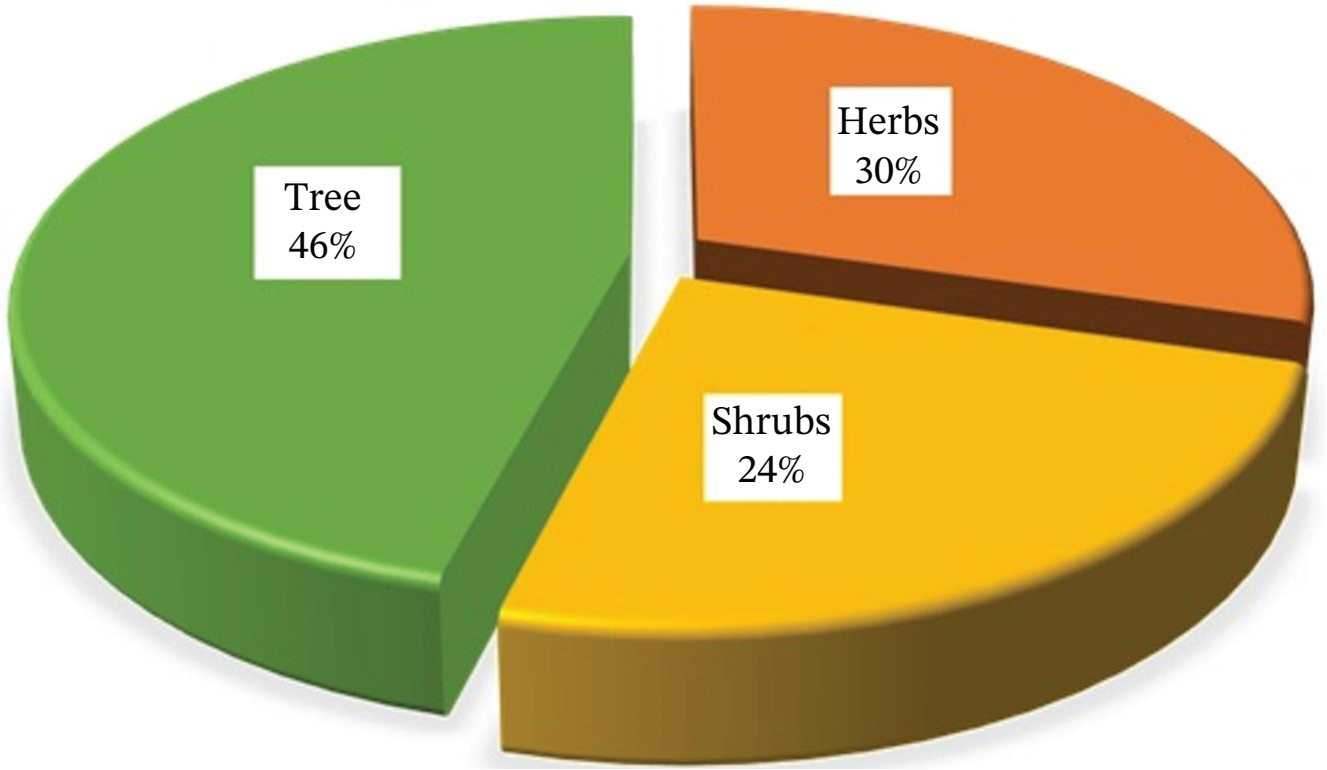
Percent share of life forms used for making herbal remedies in Tanzania.

**Figure 3:**
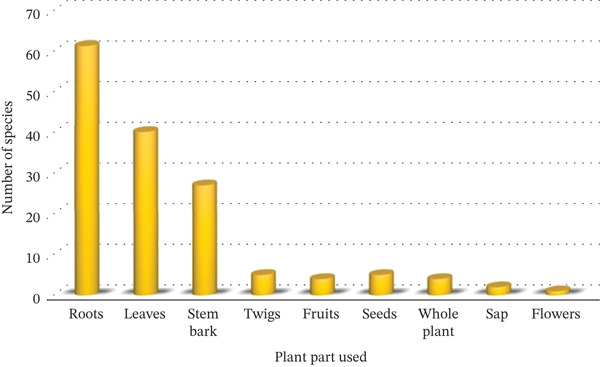
Plant parts used for making herbal remedies and the corresponding number of Fabaceae species.

Trees (46%) are the predominant source of medicinal Fabaceae plants in Tanzania, followed by herbs (30%) and shrubs (24%) (Figure 2). The plant parts used to prepare traditional medicines include roots, leaves, stem bark, root bark, bark sap, fruits, seeds, tubers, and twigs (Table 1). The most commonly used parts are roots (61 species, 63%), followed by leaves (40 species, 41%), stem bark (27 species, 28%), and twigs and seeds (five species, 5.2% each). Fruits and whole plants each account for four species (54.2%), whereas sap is derived from two species (42.1%) and flowers from just one species (1%) (Figure 3). However, exploiting roots from herbaceous MPs for traditional remedies is often unsustainable, posing significant risks to the plants and the surrounding vegetation′s root systems [40, 58]. Similarly, indiscriminate bark harvesting, especially from slow‐growing species, can lead to their extinction [59]. Conservationists emphasise that MPs are highly valued for their roots, with those subjected to intensive bark harvesting being particularly at risk of overexploitation [60]. In contrast, the use of leaves is generally promoted as a more sustainable alternative, as they can regenerate and have a less detrimental impact on the parent plant [61].

**Figure 4:**
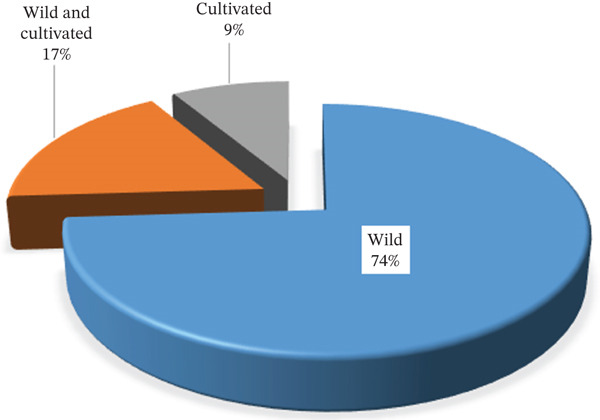
Percent share of sources of Fabaceae medicinal plant used in Tanzania.

The majority of MP resources (74%) are sourced from wild environments, followed by a combination of wild and cultivated environments (17%) and cultivated areas (9%) (Figure 4). This strong dependence on wild resources stems from their unrestricted availability and the local perception that cultivated plants are less effective. However, such practices are not sustainable in the long run. Therefore, it is crucial to protect and conserve wild environments and their associated resources to ensure a sustainable supply of MPs. Additionally, implementing macropropagation and micropropagation strategies can serve as an effective conservation measure and significantly reduce exploitation pressure on wild resources.

### 3.3. Major Ailment Categories

The ailments discussed in this review are categorised into 19 major health disorder categories (see Table 2). The majority of recorded Fabaceae species are used to treat fever and malaria (39.6%), followed by gastrointestinal disorders (29.2%), gynaecological conditions (28.1%), antivenin (21.9%), respiratory disorders (16.7%) and dermatological issues (14.6%) (Table 2). Likewise, Fabaceae species in Zimbabwe are reported to address these disorders [1], which are significant causes of mortality in various developing countries [14, 59, 62]. Notably, gastrointestinal disorders had the highest number of use records (31), followed closely by malaria and gynaecological disorders (29 records each), respiratory disorders (21 records), and antivenin (19 records). This aligns with observations in Zimbabwe, where gastrointestinal and respiratory disorders were reported to have the most extensive use records [1].

**Table 2:** Major ailment categories and number of Fabaceae species.

Ailment category	Number of species	Percentage (%)	Use records
Fever and malaria	38	39.58	29
Gastrointestinal problems	28	29.17	31
Gynaecological disorders	27	28.13	29
Antivenin	21	21.88	19
Respiratory problems	16	16.67	21
Dermatological diseases	14	14.58	9
Sexually transmitted infections (STIs)	14	14.58	6
Erectile dysfunction	12	12.50	7
Hernia	5	5.21	3
Sickle cell and anaemia	5	5.21	3
Pains (back, feet, chest, headache)	5	5.21	5
Hypertension/hypotension	3	3.13	8
Charms and rituals	2	2.08	2
Dental problems	2	2.08	3
Oedema	2	2.08	2
Diabetes mellitus	1	1.04	3
Mental problems	1	1.04	2
Neurological problems	1	1.04	3
Ophthalmological disorders	1	1.04	2

### 3.4. Phytochemistry and Pharmacological Properties of Fabaceae Species

Medicinal Fabaceae species used in traditional remedies in Tanzania are rich in major phytochemical constituents (Table 3). The majority are characterised by flavonoids (88.7%), followed by tannins (50.5%), phenolic compounds (38.1%), terpenoids (38.1%), saponins (36.1%), alkaloids (36.1%), and glycosides (16.5%) (Table 3). The findings of this review corroborate those of Maroyi [1], who reported that flavonoids are the dominant chemical constituents in the Fabaceae of Zimbabwe. Moreover, a study by Wink [209] revealed that alkaloids, phenolics, and terpenoids are the key secondary metabolites of the Fabaceae family. Most reported MPs in this review (Table 3) exhibit several proven pharmacological activities, including antimicrobial, antioxidant, antidiabetic, antifungal, anticancer, anti‐inflammatory, antiplasmodial, hepatoprotective, immunostimulatory and immunomodulatory properties. Despite the discovery of numerous secondary metabolites in the family, its MPs have attracted unduly little attention in ethnopharmacological exploration over the years. The therapeutic value of Fabaceae species is supported, as over 38.5% of the species reported in this review are commercially important, traded in both informal and formal markets locally in the country [11] and in other parts of Africa [1, 209]. This highlights the economic importance of our work, as some of these species are widely used in traditional medicine in Zimbabwe [1, 210], South Africa [211, 212], Zambia [213], Mozambique [213], and Namibia [214]. Moreover, other ethnobotanical studies have revealed that some medicinal Fabaceae plants are commercially exploited in local, regional and international markets as remedies for various ailments. Such MPs with the potential to be developed into pharmaceutical products are *E. abyssinica*, *E*. *abysinica*, *P. thonningii*, *S. mellifera*, *S. occidentalis*, *S. siamea*, *V. seyal*, *K. africana*, *C. cajan*, *A. precatorius*, *A. pilosum* and *T. indica* [11, 12, 215, 216].

**Table 3:** Evidence of major phytochemical constituents, pharmacological properties and toxicity profile of medicinal Fabaceae species.

Species	Major phytochemical constituents	Pharmacological activities	Reference	Toxicity reports
*Abrus precatorius* L.	Alkaloids, flavonoids, phenolics, terpenoids and glycosides	Abortifacient, antidiabetic, antifertility, anti‐inflammatory, antimicrobial, antioxidant, antiparasitic, antiepileptic, antiviral, antimalarial, antitumor, immunomodulatory, nephroprotective, immunostimulatory, neuromuscular effects and neuroprotective	[63, 64]	Highly toxic [65, 66]
*Afzelia quanzensis* Welw.	Alkaloids, flavonoids, phenolics, terpenoids and glycosides	Antifungal and antibacterial	[67, 68]	Non‐toxic [69]
*Aganope stuhlmannii* (Taub.) Adema	Rotenoids (deguelin, tephrosin) and flavonoids	Antimicrobial, antioxidant and insecticidal	[70]	Limited data
*Alantsilodendron pilosum* Villiers	Flavonoids, phenolics, saponins, tannins and terpenoids	Analgesic, antibacterial, antifungal, antiviral, anticonvulsant, anti‐inflammatory, antimalarial, antioxidant, hepatoprotective and neuropharmacological	[71–73]	Non‐toxic [23]
*Albizia anthelmintica* (A. Rich.) Brongn.	Alkaloids, flavonoids, phenolics, saponins and tannins	Analgesic, antibacterial, anti‐inflammatory and antioxidant	[74, 75]	Mild toxicity [76]
*Albizia gummifera* (J.F. Gmel.) http://C.A.Sm.	Glycosides, saponins and terpenoids	Antibacterial, antioxidant, antidiabetic, anthelmintic, anti‐inflammatory, hepatoprotective and cytotoxic	[77, 78]	Mild toxicity [79]
*Albizia harveyi* E. Fourn.	Flavonoids and glycosides	Antioxidant, antidiabetic and hepatoprotective	[80, 81]	Non‐toxic [23]
*Albizia lebbeck* (L.) Benth	Flavonoids (quercetin, kaempferol), saponins, tannins, alkaloids and glycosides	Anti‐inflammatory, antioxidant, antimicrobial, antiasthmatic, immunomodulatory and neuroprotective	[82, 83]	Toxic [82]
*Albizia petersiana* (Bolle) Oliv.	Saponins, flavonoids, tannins and alkaloids	Antibacterial, antioxidant, antihelmintic and wound healing; rheumatism	[84]	Limited data
*Albizia schimperiana* Oliv.	Spermine alkaloids, saponins and terpenoids	Antimicrobial, antiparasitic, cytotoxic, analgesic and antihelmintic	[86, 87]	Toxic [86]
*Albizia verrucosa* Capuron	Flavonoids, saponins, tannins and alkaloids	Antimicrobial, anti‐inflammatory, antidiarrheal and wound healing	[87]	Limited data
*Albizia versicolor* Welw. ex Oilv.	Glycosides, saponins and terpenoids	Anthelmintic and antifungal	[87, 88]	Highly toxic [89]
*Alysicarpus ovalifolius* (Schumach.) J. Léonard	Alkaloids, flavonoids, phenolics and terpenoid	Analgesic, anti‐inflammatory, antimicrobial, antiplasmodial, larvicidal, mosquitocidal, antioxidant, hepatoprotective, antiproliferative and antifertility	[90, 91]	Non‐toxic [92]
*Arachis hypogaea* L.	Alkaloids, phenolics and saponins	Antioxidant, antiobesity, antidiabetic, antihypertensive and hypolipidemic	[93, 94]	Non‐toxic [95, 96]
*Bauhinia tomentosa* Vell.	Glycosides, terpenoids, saponin and flavonoids	Chemoprotective anticancer activity, anti‐inflammatory, antidiabetic and antioxidant	[97]	Non‐toxic [98]
*Bobgunnia madagascariensis* (Desv.) J.H.Kirkbr. & Wiersema	Flavonoids, saponins and tannins	Antibacterial	[99]	Highly toxic [100, 101]
*Brachystegia boehmii* Taub.	Tannins	Antibacterial, anti‐inflammatory and antioxidant	[102]	Non‐toxic [103]
*Brachystegia spiciformis* Benth.	Flavonoids and tannins	Antibacterial	[104, 105]	Non‐toxic [23]
*Burkea africana* Hook.	Flavonoids, glycosides, and saponinstannins and triterpenes	Analgesic, antibacterial, antiviral, anticholinesterase, anti‐inflammatory and antioxidant	[106, 107]	Non‐toxic [108]
*Cajanus cajan* (L.) Huth	Flavonoids and phenolics	Antioxidant and anti‐inflammatory	[109]	Non‐toxic [110]
*Cassia abbreviata* Oliv.	Anthocyanins, phenolics and tannins	Abortifacient, antidiabetic, anti‐inflammatory, antimicrobial, antiviral, antioxidant and hepatoprotective	[111, 112]	Non‐toxic [23]
*Cassia burttii* Baker f.	Flavonoids, anthraquinones, alkaloids, tannins, phenols and steroids	Antimicrobial, antioxidant, laxative, hepatoprotective and anti‐inflammatory	[113]	Limited data
*Chamaecrista absus* (L.) H.S. Irwin and Barneby	Alkaloids, flavonoids, glycosides, tannins, and terpenoids	Antihypertensive, antifertility, antifungal, anti‐inflammatory, antihyperglycemic, antiglycation, antibacterial activity, *α*–amylase inhibitory, antioxidant and reducing activity	[114, 115]	Mild toxicity [116]
*Chamaecrista mimosoides* (L.) Greene	Flavonoids, anthraquinones, tannins, alkaloids and glycosides	Antiepileptic, antimicrobial, antioxidant and anti‐inflammatory	[83]	Non‐toxic [117]
*Dalbergia malangensis* E.C. Sousa	Isoflavonoids, neoflavonoids, quinones and tannins	Antimicrobial, anti‐inflammatory, antimalarial and antioxidant	[118]	Toxic [119]
*Dalbergia melanoxylon* Guill. and Perr.	Alkaloids, flavonoids, glycosides and tannins	Analgesic, anti‐inflammatory, antimicrobial, antiviral, antioxidant and antipyretic	[120, 121]	Non‐toxic [122]
*Dalbergia nitidula* Baker	Flavonoids	Antibacterial, antioxidant and cytotoxic	[123, 124]	Non‐toxic
*Delonix elata* (L.) Gamble	Phenolics and flavonoids	Antioxidant and prophylactic	[125]	Non‐toxic [126]
*Dolichos kilimandscharicus* Taub.	Flavonoids and saponins	Antibacterial, anticancer, antiproliferative and cytotoxic	[127, 128]	Limited data
*Dolichos oliveri* Schweinf.	Flavonoids, saponins, tannins, terpenoids and alkaloids	Antischistosomal, antimicrobial, antioxidant and anti‐inflammatory	[129, 130]	Limited data
*Entada abyssinica* Steud. Ex A. Rich.	Flavonoid and terpenoids	Antibacterial, anti‐inflammatory, antioxidant, antiplasmodial, antiproliferative, antifungal, antimycobacterial, antidiarrheal, anti‐HIV 1, antidiabetic, and antiobesity antioxidant, antiplasmodial, antiproliferative, antifungal, antimycobacterial, antidiarrheal, anti‐HIV 1, antidiabetic and antiobesity	[131, 132]	Mild toxicity [133]
*Entada leptostachya* Harms	Flavonoids, tannins, and saponins	Antioxidant and antimicrobial	[134]	Limited data
*Eriosema parviflorum* E. Mey	Phenolics and flavonoids	Aphrodisiac, antiosteoporosis, hypolipidemic, antidiabetic, antidiarrheal, antimicrobial, antioxidant, anthelmintic, anticancer and acetylcholinesterase inhibitory	[135]	Limited data
*Eriosema psoraleoides* (Lam.) G. Don.	Phenolics and flavonoids	Aphrodisiac, estrogenic, antiosteoporosis, hypolipidemic, antidiabetic, antidiarrheal, antimicrobial, antioxidant, anthelmintic, anticancer and acetylcholinesterase inhibitory	[135]	Limited data
*Erythrina abysinica* Lam.	Alkaloids, flavonoids and terpenoids	Antibacterial, antifungal, antiviral, antidiabetic, anti‐inflammatory, antioxidant, antiplasmodial, antiproliferative and hepatoprotective	[132, 136, 137]	Mild toxicity [132]
*Erythrina haerdii* Verdc.	Alkaloids and flavonoids	Sedative and anti‐inflammatory	[137]	Toxic [137]
*Erythrina sacleuxii* Hua	Flavonoids, terpenoids, phenolics, triterpenes and coumarins	Antiplorifelative	[139, [137] 142]	Non‐toxic [138]
*Erythrina schliebenii* Harms	Flavonoids, terpenoids, phenolics, triterpenes and coumarins	Antiplorifelative, antifungal and cytotoxic	[137, 139]	Limited data
*Faidherbia albida* (Delile) A. Chev.	Alkaloids, flavonoids, phenolics, tannins, saponins and terpenoids	Antimicrobial, antioxidant, antischistosomal and antifungal	[140]	Non‐toxic [141]
*Guilandina volkensii* (Harms) G.P. Lewis	Terpenoids and flavonoids	Antimalarial and antimicrobial	[142]	Limited data
*Indigofera arrecta* Hochst ex A. Rich.	Alkaloids, flavonoids, glycosides, phenols, saponins, tannins and terpenoids	Antiplasmodial, antimicrobial, antinecrotic, cytotoxic, anti‐inflammatory, hepatoprotective, neuroprotective and renoprotective	[143, 144]	Mild toxicity
*Indigofera drepanocarpa* Taub.	Alkaloids, flavonoids, phenolics and terpenes	Antimicrobial and anti‐inflammatory	[145]	Limited data
*Indigofera lupatana* Baker f.	Flavonoids, alkaloids, phenolics and terpenoids	Antimicrobial, antioxidant and cytotoxic activity	[146]	Toxic [146]
*Indigofera rhynchocarpa* Welw. ex Baker	Flavonoids, terpenoids, indole alkaloids and glycosides	Antimicrobial, anti‐inflammatory and antioxidant	[145]	Limited data
*Indigofera suffruticosa* Mill.	Indigotin and flavonoids	Antidiabetic, hepatoprotective, nti‐inflammatory, antibacterial, antifungal, antioxidative, antitumor, antimutagenic, anticonvulsant, gastroprotective and hepatoprotective activities	[147]	Limited data
*Indigofera swaziensis* Bolus	Flavonoids and alkaloids	Antimicrobial	[145]	Limited data
*Indigofera volkensii* Taub.	Flavonoids and alkaloids	Antimicrobial	[145]	Limited data
*Isoberlinia angolensis* (Welw. ex Benth.) Hoyle and Brenan	Tannins, flavonoids, saponins and alkaloids	Antioxidant, anti‐inflammatory and antimicrobial	[148]	Limited data
*Julbernardia globiflora* (Benth.) Troupin	Tannins	Anticancer	[104]	Non‐toxic
*Kotschya africana* Endl.	Flavonoids, phenolics, alkaloids and terpenoids	Antimicrobial, antifungal, antiviral and mosquito larvicidal	[149]	Limited data
*Lonchocarpus capassa* Rolfe	Terpenoids and Flavanoids	Antioxidant, antifungal and cytotoxic	[150]	Mild toxicity [150]
*Macrotyloma axillare* (E. Mey) Verdc.	Flavonoids and tannins	Antioxidant and forage value	[151]	Non‐toxic [151]
*Millettia lasiantha* Dunn	Rotenoids and flavonoids	Insecticidal and antimicrobial	[152]	Toxic [152]
*Millettia usaramensis* Taub.	Flavonoids, phenolics, saponins, alkaloids and terpenoids	Antibacterial, antitumour, insecticidal, pesticidal, antispasmodial and chemopreventive	[153]	Mild toxicity [154]
*Mucuna pruriens* (L.) DC	Phenolics and flavinoids	Antiparkinson, antioxidant, antivenom, antimicrobial and antineuroprotective	[155]	Mild toxicity [156]
*Mundulea sericea* (Willd.) A. Chev.	Flavonoids, phenolics, saponins and tannins	Analgesic, antibacterial, antifungal, antioxidant and insecticidal	[157]	Highly toxic
*Ormocarpum kirkii* S. Moore	Flavonoids and terpenoids	Antibacterial, antifungal, antimalarial, antiplasmodial and cytotoxicity	[158]	Non‐toxic [159]
*Ormocarpum trachycarpum* (Taub.) Harms	Flavonoids and tannins	Antimicrobial	[160]	Limited data
*Pericopsis angolensis* (Baker) Meeuwen	Flavonoids, saponins and tannins	Antimicrobial	[99]	Limited data
*Philenoptera bussei* (Harms) Schrire	Isoflavones and tannins	Antioxidant	[161]	Limited data
*Piliostigma thonningii* (Schumach.) Milne‐Redh.	Alkaloids, flavonoids, saponins, tannins and terpenes	Analgesic, anthelminthic, antibacterial, antiviral, antimalarial, anti‐inflammatory, antileishmanial, antioxidant, antipyretic and immunomodulatory	[162, 163]	Non‐toxic [163]
*Pleurolobus salicifolius* (Poir.) H. Ohashi and K. Ohashi	Flavonoids and phenolics	Antimicrobial	[151]	Limited data
*Pseudovigna argentea* (Willd.) Verdc.	Flavonoids, tannins	Antioxidant		Limited data
*Pterocarpus angolensis* DC.	Flavonoids, phenolics and terpenoids	Antibacterial, antifungal, antiviral, anticancer, anti‐inflammatory, antioxidant and wound healing	[164, 165]	Non‐toxic [166]
*Pterocarpus tinctorius* Welw.	Phenolics, flavonoids, tannins and saponins	Antibacterial	[167]	Limited data
*Senegalia polyacantha* (Willd.) Seigler and Ebinger	Tannins and saponins	Antimicrobial and anti‐inflammatory	[168]	Limited data
*Senegalia brevispica* (Harms) Siegler and Ebinger	Alkaloids, flavonoids, terpenoids, phenolics, tannins and saponins	Antibacterial, Anti‐inflammatory, Antihypertensive, antiplatelet, Hypoglycemic, Antiatherosclerotic, Analgesic and anticancer	[169]	Limited data
*Senegalia mellifera* (Vahl) Siegler and Ebinger	Flavonoids, glycosides, phenolics, saponins, tannins and terpenoids	Antibacterial, antifungal and cytotoxicity	[170, 171]	Non‐toxic [172]
*Senegalia nigrescens* (Oliv.) P.J.H. Hurter	Flavonoids and triterpenoids	Antimicrobial, antioxidant and cytotoxicity	[173]	Non‐toxic [174]
*Senegalia pentagona* (Schumach.) Hook. f.	Tannins and flavonoids	Antimicrobial	[168]	Limited data
*Senegalia polyacantha* (Willd.) Seigler and Ebinger	Flavonoids, tannins, saponins, terpenoids and alkaloids	Antimicrobial, anti‐inflammatory and antiplasmodial	[175]	Limited data
*Senegalia senegal* (L.) Britton	Gum arabic (polysaccharides)	Demulcent and prebiotic	[168]	Non‐toxic [168]
*Senna alata* (L.) Roxb.	Anthraquinones	Antifungal and laxative	[176]	Toxic [176]
*Senna didymobotrya* (Fresen.) H.S. Irwin and Barneby	Alkaloids, flavonoids, phenolics, saponins, tannins and terpenoids	Antibacterial	[177, 178]	Toxic [159]
*Senna obtusifolia* (L.) H.S. Irwin and Barneby	Glycosides, flavonoids and phenolics	Antidiabetic, antimicrobial, anti‐inflammatory, hepatoprotective and neuroprotective	[179]	Non‐toxic [180]
*Senna occidentalis* (L.) Link	Alkaloids, flavonoids, and saponins	Antibacterial, antifungal, anticancer, antidiabetic, anti‐inflammatory, antimutagenic, antiprotozoal and hepatoprotective	[181, 182]	Toxic [183]
*Senna siamea* (Lam.) H.S. Irwin and Barneby	Flavonoids and terpenoids	Anti‐inflammatory, antimicrobial, antioxidant, antidiabetic, antiallergic, hepatoprotective, neuroprotective and anti‐Alzheimer′s disease activities	[184, 185]	Non‐toxic [186]
*Senna singueana* (Delile) Lock	Alkaloids, phenols, saponins, tannins and terpenes	Antimalarial, antinociceptive, antioxidant, hepatoprotective and trypanocidal	[182]	Non‐toxic [159, 187]
*Sesbania microphylla* Harm.	Flavonoids and tannins	Antioxidant	[188]	Limited data
*Sesbania sesban* (L.) Merr.	Isoflavones and tannins	Antioxidant and forage	[188, 189]	Non‐toxic [188, 189]
*Tamarindus indica* L.	Tannins	Antibacterial, antifungal, antiviral, antidiabetic, anti‐inflammatory, antinematodal, antioxidant, cytotoxic and molluscicidal	[190]	Non‐toxic [191]
*Tephrosia aequilata* Baker	Flavonoids	Antiveneral	[192]	Toxic [193]
*Tephrosia luzoniensis* Vogel	Rotenoids, flavonoids; flavonoids; chalcones; phenolic acids; and tannins	Insecticidal, antimicrobial, anti‐inflammatory and pesticidal	[194, 195]	Toxic [194, 195]
*Tephrosia purpurea* (L.) Pers.	Flavonoids and rotenoids	Hepatoprotective, antioxidant and anti‐inflammatory	[195]	Limited data
*Tephrosia vogelii* Hook.f.	Rotenoids, flavonoids; flavonoids; chalcones; phenolic acids; and tannins	Insecticidal, antimicrobial	[194, 196]	Toxic [194, 196]
*Vachelia tortilis* (Forssk.) Galasso and Banfi	Flavonoids	Antibacterial, antidiabetic, antiparasitic, cytotoxic and anti‐inflammatory	[197]	Mild toxicity [198]
*Vachellia drepanolobium* (Harms ex Y. Sjöstedt) P.J.H. Hurter	Tannins and flavonoids	Antimicrobial	[199]	Limited data
*Vachellia gerrardi* (Benth.) P.J.H. Hurter	Tannins and saponins	Antioxidant	[199]	Limited data
*Vachellia hockii* (De W.) Seigler and Ebinger	Alkaloids, flavonoids, tannins, saponins and terpenoids	Antibacterial, antifungal, antimalarial, antiviral, antiparasitic, and antidiabetic properties. It is also used to treat opportunistic infections in HIV/AIDS, anaemia, blood cleansing, cough and hernia	[159]	Limited data
*Vachellia nilotica* (L.) P.J.H. Hurter and Mabb	Alkaloids, flavonoids and tannins	Inhibition of acetylcholinesterase, anthelmintic, antibacterial, anticancer, antihypertensive, anti‐inflammatory, antioxidant and antiplatelet	[200]	Mild toxicity [180, 198]
*Vachellia robusta* (Burch.) Kayl. and Boatwr.	Tannins and flavonoids	Antimicrobial	[199]	Limited data
*Vachellia seyal* (Delile) P.J.H. Hurter	Flavonoids, saponins and phenolics	Anticarcinogenic, antimalarial, antisickle‐cell anaemia, antibacterial, antidiabetic, immunomodulatory, Antihypercholesterolimic, anti‐inflammatory, antioxidant, Hepatoprotective and antiulcerative	[201]	Mild toxicity [198]
*Vachellia sieberiana* (DC.) Kyal. and Boatwr.	Flavonoids, glycosides, phenolics, saponins and tannins	Antibacterial and anticancer	[202]	Mild toxicity [198, 203]
*Vachellia zanzibarica* (S. Moore) Kyal. and Boatwr.	Tannins and flavonoids	Antimicrobial	[199]	Limited data
*Vigna radiata* (L.) R. Wilczek	Isoflavones and phenolics	Antioxidant and antidiabetic	[204]	Non‐toxic [204]
*Vigna unguiculata* (L.) Walp.	Flavonoids and phenolics	Acetylcholinesterase inhibition, anthelmintic, antibacterial, antifungal, antiviral, antidiabetic, anti‐inflammatory, antioxidant, antinociceptive and hypocholesterolaemic	[205, 206]	Non‐toxic [207]
*Zenkerella grotei* (Harms) J. Léonard	Flavonoids, tannins, alkaloids, saponins, phenolic acids and Coumarins	Limited pharmacological data	[208]	Limited data

Regarding toxicity profiles, 37.0% of the surveyed MPs were associated with insufficient or limited toxicological data, thereby restricting comprehensive risk evaluation. In contrast, 33.0% were consistently documented as nontoxic across available studies. The remaining 30.0% exhibited variable toxicity outcomes, with adverse effects shown to be dose‐dependent, underscoring the critical importance of dosage considerations in both pharmacological practice and ethnomedicinal applications [217, 218]. Therefore, these findings highlight a pressing need for evidence‐based regulatory frameworks and standardized toxicological assessments to guide safe integration of MPs into public health systems and policy development.

## 4. Conclusion

The Fabaceae family contributes significantly to Tanzania′s traditional healthcare, with 97 medicinal taxa offering notable therapeutic potential. To ensure safe integration into public health systems, urgent action is required to establish evidence‐based regulatory frameworks and standardized toxicological protocols. Moreover, aligning ethnomedicine with modern research will strengthen cultural continuity, improve health outcomes, and create pathways for pharmaceutical innovation.

## Author Contributions

D.S.K. conceived, designed the study, collected, analysed and interpreted the data, and wrote the article.

## Funding

No funding was received for this manuscript.

## Conflicts of Interest

The author declares no conflicts of interest.

## Data Availability

The data that support the findings of this review are available from the corresponding author upon reasonable request.
